# Hypoxia upregulating ACSS2 enhances lipid metabolism reprogramming through HMGCS1 mediated PI3K/AKT/mTOR pathway to promote the progression of pancreatic neuroendocrine neoplasms

**DOI:** 10.1186/s12967-024-04870-z

**Published:** 2024-01-23

**Authors:** Danyang Gu, Mujie Ye, Guoqin Zhu, Jianan Bai, Jinhao Chen, Lijun Yan, Ping Yu, Feiyu Lu, Chunhua Hu, Yuan Zhong, Pengfei Liu, Qibin He, Qiyun Tang

**Affiliations:** 1 Department of Geriatric Gastroenterology, Institute of Neuroendocrine Tumor, Neuroendocrine Tumor Center, Jiangsu Province Hospital, The First Affiliated Hospital of Nanjing Medical University, Nanjing Medical University, No. 300 Guangzhou Road, Nanjing, 210029 China; 2 Department of Gastroenterology, Jiangyin People’s Hospital, Jiangsu, China; 3 Department of Gastroenterology, The Affiliated Jiangning Hospital of Nanjing Medical University, Jiangsu, China

**Keywords:** Hypoxia, ACSS2, Lipid metabolism reprogramming, HMGCS1, PI3K/AKT/mTOR pathway, Pancreatic neuroendocrine neoplasms

## Abstract

**Background:**

Pancreatic neuroendocrine neoplasms (pNENs) are relatively rare. Hypoxia and lipid metabolism-related gene acetyl-CoA synthetase 2 (ACSS2) is involved in tumor progression, but its role in pNENs is not revealed. This study showed that hypoxia can upregulate ACSS2, which plays an important role in the occurrence and development of pNENs through lipid metabolism reprogramming. However, the precise role and mechanisms of ACSS2 in pNENs remain unknown.

**Methods:**

mRNA and protein levels of ACSS2 and 3-hydroxy-3-methylglutaryl-CoA synthase1 (HMGCS1) were detected using quantitative real-time PCR (qRT-PCR) and Western blotting (WB). The effects of ACSS2 and HMGCS1 on cell proliferation were examined using CCK-8, colony formation assay and EdU assay, and their effects on cell migration and invasion were examined using transwell assay. The interaction between ACSS2 and HMGCS1 was verified by Co-immunoprecipitation (Co-IP) experiments, and the functions of ACSS2 and HMGCS1 in vivo were determined by nude mouse xenografts.

**Results:**

We demonstrated that hypoxia can upregulate ACSS2 while hypoxia also promoted the progression of pNENs. ACSS2 was significantly upregulated in pNENs, and overexpression of ACSS2 promoted the progression of pNENs and knockdown of ACSS2 and ACSS2 inhibitor (ACSS2i) treatment inhibited the progression of pNENs. ACSS2 regulated lipid reprogramming and the PI3K/AKT/mTOR pathway in pNENs, and ACSS2 regulated lipid metabolism reprogramming through the PI3K/AKT/mTOR pathway. Co-IP experiments indicated that HMGCS1 interacted with ACSS2 in pNENs. Overexpression of HMGCS1 can reverse the enhanced lipid metabolism reprogramming and tumor-promoting effects of knockdown of ACSS2. Moreover, overexpression of HMGCS1 reversed the inhibitory effect of knockdown of ACSS2 on the PI3K/AKT/mTOR pathway.

**Conclusion:**

Our study revealed that hypoxia can upregulate the lipid metabolism-related gene ACSS2, which plays a tumorigenic effect by regulating lipid metabolism through activating the PI3K/AKT/mTOR pathway. In addition, HMGCS1 can reverse the oncogenic effects of ACSS2, providing a new option for therapeutic strategy.

**Supplementary Information:**

The online version contains supplementary material available at 10.1186/s12967-024-04870-z.

## Introduction

Neuroendocrine neoplasms (NENs) are a group of tumors that originate from neuroendocrine cells and are prevalent in the gastrointestinal tract and pancreas [1]. In the pancreas, NENs were first identified by Langerhans in 1869 [2], and although the incidence of pNENs is relatively low, accounting for only 1–2% of pancreatic tumors, their incidence is increasing yearly [3]. pNENs have been classified as functional or nonfunctional based on whether or not they release symptom-producing hormones, and the majority of pNENs are nonfunctional. Functional pNENs are uncommon and can release different hormones, such as insulin, glucagon, gastrin, vasoactive intestinal peptide, and growth inhibitors and so on [4]. So far, more and more studies have focused on the clinical and basic research on pNENs, and their treatment also tends to be comprehensive [5]. Current studies have shown that pNENs are mainly affected by 3 molecular pathways, namely MEN1 inactivation, DAXX/ATRX mutations, and alterations in the mammalian target protein pathway of rapamycin(mTOR), and that patients with pNENs with MEN1 and DAXX/ATRX mutations have a better prognosis, whereas patients with pNENs with mTOR mutations have a worse prognosis [6]. Although the exploration of pNENs has made some progress, the research on pNENs needs to be further improved.

Hypoxia is a common and important feature of tumors and is closely associated with tumor progression, increased tumor aggressiveness, and low tumor survival [7]. When a cell is deprived of oxygen, it adapts to hypoxia by reprogramming many of its genes for energy metabolism, as do tumor cells. Although a large number of studies have emerged on the regulation of metabolism by hypoxia-inducible factors (HIF), it is only in recent years that the effects of hypoxia and HIF on lipid metabolism have come to the forefront of research [8]. Lipids are energy-rich compounds that can be degraded to provide ATP and play a role in cellular bioenergetics. Therefore, the regulation of lipid synthesis, uptake and degradation is essential for the maintenance of cellular physiology, and lipid metabolism is increasingly recognized as a potential therapeutic target for tumors [9].

The only carbon source and precursor for fatty acid biosynthesis in mammalian cells is acetyl coenzyme A (acetyl-CoA). The synthetic precursors of acetyl-CoA are mainly citrate and acetate. Citrate generates acetyl-CoA under the action of ATP citrate lyase (ACLY), while acetate generates acetyl-CoA under the action of acetyl-CoA synthetase (ACSS) [10]. There have been more studies to prove the role of ACLY in inhibiting the growth of tumors and only a few studies have explored the potential role of ACSS in tumors, while its study in NENs has not been reported. ACSS includes three family members: ACSS1, ACSS2, ACSS3, among which ACSS2 has been studied more in tumors, and it plays different roles in different tumors.

In our study, the aim is to investigate the effect of hypoxia conditions on the lipid metabolism related gene ACSS2, and the role it plays in the growth and survival of pNENs cells, for exploring potential therapeutic strategies for pNENs.

## Materials and methods

### Cell culture

The Human Pancreatic Nestin-Expressing ductal cells line (HPNE) was purchased from the ATCC (CBP60857). The human pNENs were obtained from the JCRB cell band (JCRB0183), and the other pNENs cell is named PNET that we isolated primary human pNENs cell from the pNENs tissues of patients diagnosed with pNENs [11]. HPNE and QGP-1 cells were cultured in RPMI-1640 medium (Gibco) and PNET cells were cultured in McCoy’s 5A medium (Gibco). All cells were maintained in a humidified incubator with 21% O_2_, 5% CO_2_ at 37 °C, and cells under hypoxia were cultured in a humidified incubator with1% O_2_, 5% CO_2_ at 37 °C.

### Construction of stable transmissible cells

ACSS2 knockdown lentiviral and HMGCS1 overexpression (OE) lentiviral were designed and synthesized by GenePharma (Suzhou, China). HIF-1a, ACSS2 overexpression plasmids were constructed in the PLVX vector by Genomeditech. HIF-1a and ACSS2 knockdown plasmids were constructed in the PLKO1 vector by Genomeditech, 293 T cells were used for lentivirus packaging. Stable transmissible cells were screened with puromycin and verified by qRT-PCR and WB.

### qRT-PCR

Total RNA was extracted by Trizol reagent (Life), and genomic DNA was removed with 5 × g DNA digester (Yeasen) with the reaction carried out at 42 ℃ for 2 min, and then adding 4xHifair^®^ III SuperMix plus (Yeasen) at 37 ℃ for 15 min and 85 ℃ for 5 s for reverse transcription to synthesize cDNA. qRT-PCR was performed using a Roche instrument and a SYBR Green PCR master mix(Yeasen) with the following steps:95 ℃ for 5 min, 35 cycles at 95 ℃ for 30 s, 58 ℃ for 30 s, 72 ℃ for 30 s, ACTIN was used as an internal control. The primer sequences are shown in Additional file 2: Table S1.

### WB

Cells were lysed in NP40 buffer (Beyotime) containing 1% 100 mM PMSF (Beyotime) for 30 min, and then boiled under 1Xloading buffer at 105 ℃ for 10 min. An equal amount of protein was loaded on 10% SDS-PAGE gels and electrically transferred to a nitrocellulose filter membrane after separation. The membrane was blocked with 8% de-skimmed milk (4 g milk powder with 50 ml TBS Tween-20 buffer) for 1–2 h and then incubated with primary antibodies at 4 °C overnight. Then, after using TBS-Tween washing membrane three times for 10 min each time, the band was incubated with anti-rabbit IgG or anti-mouse IgG at room temperature for 60 min. All antibodies used are shown in Additional file 2: Table S2. The images were created by adding enhanced chemiluminescence (NCM Biotech) with image laboratory software.

### CUT–RUN

The CUT&RUN assay was conducted using Hyperactive pG-MNase CUT&RUN Assay Kit for qPCR (Vazyme, HD101). Briefly, 1 × 10^6^ primary hypoxia and normoxia pNENs cells were collected and washed once with 500 μl wash buffer before they were bound to ConA beads for 10 min at room temperature. After that, cells were incubated with 1 μg HIF-1a antibody at 4 °C overnight. Anti-mouse IgG was added and incubated for 1 h at 25 °C the next day. Then cells were washed three times with DIG-wash buffer (Vazyme, HD101) and incubated with 100 μl pG-MNase Enzyme premix (Vazyme, HD101) for 1 h at 4 °C. Similarly, cells were washed three times with DIG-wash buffer, resuspended in fragmentation buffer and incubated at 37 °C for 30 min. Add 100 μl of stop buffer to stop fragmentation and DNA was extracted by using column-based extraction reagents. DNA was eluted with double-distilled water. qPCR amplifications were performed on the ABI 7500Thermocycler (Applied Biosystems) in 20ul reaction volumes containing DNA, primers, and ChamQ Universal SYBR qPCR Master Mix. The expression of ACSS2 was calculated by a standard curve method. The sequence of ACSS2 primers was as follows: Forward 5ʹ-TCTGGTAGGGTCCACGTCTC-3ʹ, Reverse 5ʹ-TGGACTCCACTAAGGGAGCA-3ʹ.

### Immunofluorescence

A total of 2 × 104 cells were plated in 24-well plates and incubated with 200 μl hybrid solution overnight, Next, cells were washed with PBS-Tween (PBST) threes times and fixed at 4% paraformaldehyde. Cells were incubated with 0.5% Triton X-100 for 15 min and then blocked with goat serum for 30 min. After blocking, using primary antibody against ACSS2 incubated for 1-h.After cells were washed by PBST three times and then coupled with 1-h incubation with Coralite488-conjugated goat anti-Rabbit IgG (Proteintech) at room temperature. Next, cells were stasinedwith Hoechst33342 for 30 min, the images were rinsed randomly using a fluorescence microscope (Olympus Optics).

### Cell proliferation

For the cell counting kit 8 (CCK-8) assay(Yeason), 5 × 10^3^ pNENs cells were inoculated into 96-well plates containing 100 µl of CCK8 reagent and treated with 10ul of CCK8 reagent for 2 h, the cell quantity was detected using a microplate reader. For the colony formation assay, 1 × 10^3^ cells were inoculated in 6-well plates and cultured in complete medium for 2 weeks, fixed with 4% paraformaldehyde for 20 min, 0.25% crystal violet staining for 30 min. For the EdU assays, cells were inoculated in 96-well plates and treated with 50 µM EdU for 2 h at 37 °C, and then cells were fixed in 4% paraformaldehyde. The cells were incubated with 1XApollo reaction cocktail (RiboBo) for 30 min after being permeabilized with 0.5% Triton-X, then DNA was stained with Hoechst33342, and finally the images were observed by fluorescence microscopy.

### Cell migration and invasion assay

In total, 5 × 10^5^ QGP-1, 2 × 10^4^ PNET cells were inoculated in the transwell chambers (Corning) for migration experiments and 1 × 10^6^ QGP-1, 4 × 10^4^ PNET cells were inoculated in the chambers for invasion experiments. Cells were inoculated in the upper chamber containing 200 µl of serum-free medium and 600 µl of 30% fetal bovine serum. The cells were cultured for 48 h and then fixed with 4% paraformaldehyde for 30 min, then stained with 2% crystal violet for 30 min. Cells on the upper sides were wiped with a swab, while cells on the lower sides were randomly imaged under a microscope.

### Co-IP

Incubate an equal amount of QGP-1 cell lysate with either plain IgG antibody or anti-ACSS2 antibody or anti-HMSCS1 antibody and then rotate at 4 °C for 2 h. Then add 50 μl of Protein A/G agarose beads (Beyotime) to each tube and rotate at 4 °C overnight. The beads were then rinsed 5 times with RIPA and boiled for 5 min. The ACSS2 or HMGCS1 bands were then detected by WB.

### Nile red staining assay

Nile red staining was used to detect cell lipid droplet. Transgenic QGP-1 and PNET cells were inoculated into 96-well plates. After cells were adhered to the wall, they were fixed with 4% paraformaldehyde for 30 minutes and then stained with Nile red (1 mg/ml) and Hoechst33342 for 20 min of incubation at room temperature. Images were randomly captured under the microscope.

### Quantitation of triglyceride, free fatty acids and total cholesterol

Triglyceride concentration was measured using the Adipogenesis Detection Assay (Abcam). Free fatty acids concentration was measured using the Free Fatty Acid Assay Kit (Abbkine) and total cholesterol concentration was measured using the Total Cholesterol Assay Kit (Abbkine).

### Animal experiments

In a mouse xenotransplantation model, QGP-1 cells (2 × 106) with ACSS2 knockdown group, overexpression of HMGCS1 in ACSS2 knocked down group, and PLKO1 group were subcutively injected into male BALB/c nude mice (4–5 weeks old). After 5 weeks, the mice were killed by euthanasia, the tumors were removed, and then the tumors were weighed, measured in size (width and length), photographed, and fixed or frozen in 4% paraformaldehyde for further analysis. The tumor volume (V) was calculated by the formula :V = width^2^ × length/2. The obtained tumors were accurately weighed to calculate the inhibition rate (TIR) by the following formula: TIR (%) =(W_control_ − W_sample_)/W_control_ × 100. The animal experiment procedures were approved by the Animal care and Use Committee of Nanjing Medical University.

### Immunohistochemistry

Tumor tissues were fixed with 4% formaldehyde and embedded with paraffin. After cutting and dewaxing, the slices were incubated in citrate antigen repair buffer, closed with 3% bovine serum albumin blocking buffer, and then incubated with primary antibody at 4 °C overnight. Then, the slices were washed three times and incubated with secondary antibody at room temperature for 1 h. DAB color development solution was added and hematoxylin staining was performed. Finally, the slices were dehydrated and images were taken randomly by Optical microscope (CIC: XSP-C204)

### Statistical analysis

All experiments were independently repeated at least three times. All data are expressed as mean ± standard deviation. The means between groups were compared using the unpaired or paired student’s t test. All statistical analysis and experimental charts were performed using GraphPad software and *p*-value < 0.05 was significant.

## Results

### Hypoxia upregulated ACSS2 in pNENs and ACSS2 was highly expressed in pNENs

**Fig. 1:**
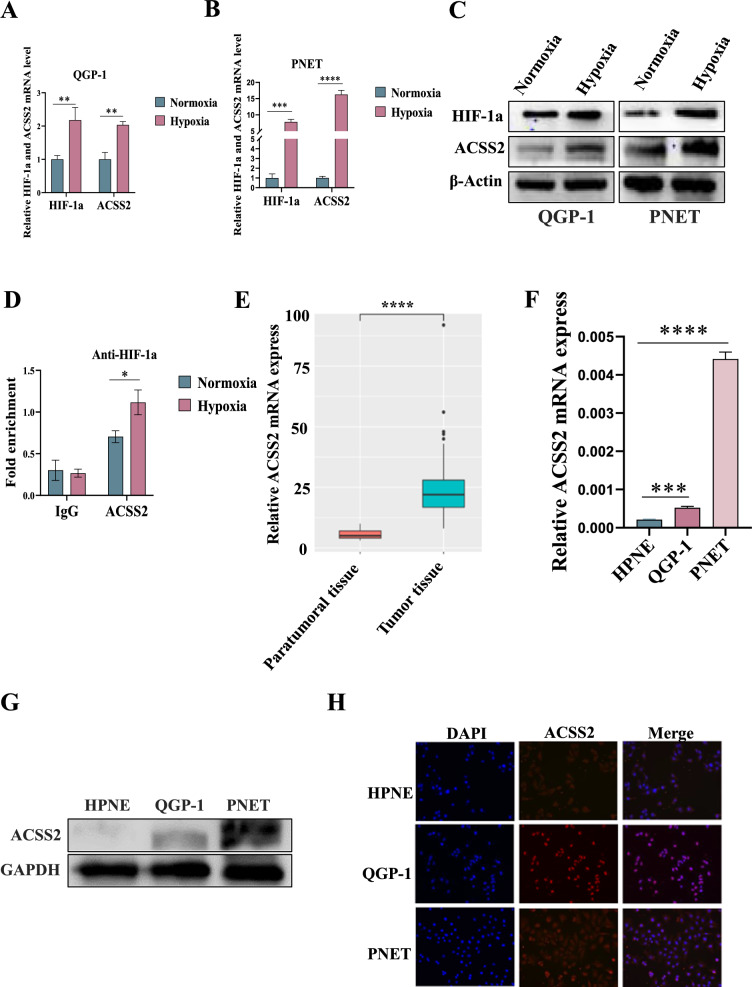
Hypoxia upregulated ACSS2, which is highly expressed in pNENs. **A**–**C** qRT-PCR and WB indicated that hypoxia upregulated ACSS2 expression in pNENs cells. **D** CUT-RUN showed increased binding of HIF-1a to ACSS2 after hypoxia in pNENs cells. **E** EGA database showed higher ACSS2 expression in pNENs tumor tissues than pNENs paraneoplastic tissues. **F**, **G**, **H** qRT-PCR, WB and immunofluorescence indicated that ACSS2 expression was higher in pNENs cells than in HPNE. *p < 0.05; **p < 0.01, ***p < 0.001, ****p < 0.0001

To investigate the role of hypoxia on the lipid metabolism-related gene ACSS2, we subjected pNENs cells QGP-1 and PNET to hypoxia treatment, and the results showed that the expression of ACSS2 in pNENs cells after hypoxia was higher than that in pNENs cells cultured in normoxia (Fig. 1A–C). Meanwhile, the binding of ACSS2 to HIF-1a was elevated after hypoxia in pNENs cells as demonstrated by CUT-RUN assay (Fig. 1D). After pNENs cells were hypoxic, CCK8 assay and colony formation assay showed that hypoxia promoted the proliferation of pNENs cells (Additional file 1: Fig. S1A–D), and EdU assay also confirmed the promotional effect of hypoxia on the proliferation of pNENs cells (Additional file 1: Fig. S1E, F), and transwell assay showed that hypoxia could promote the migration and invasion of pNENs cells (Additional file 1: Fig. S1G, H). To further investigate the role of hypoxia in pNENs cells, stable overexpression of HIF-1a and knockdown of HIF-1a QGP-1, PNET cell lines were constructed with lentiviruses and the transfection efficiency was confirmed, and we also found that the expression of ACSS2 was elevated after HIF-1a overexpressed and decreased after HIF-1a knocked down (Additional file 1: Figs. S2A–C, S3A–C). Cell proliferation assay and transwell assay found that overexpression of HIF-1a promoted the proliferation, migration and invasion of pNENs cells (Additional file 1: Fig. S2D–K). Meanwhile, knockdown of HIF-1a inhibited the proliferation, migration and invasion of pNENs cells (Additional file 1: Fig. S3D–K). Since pNENs are relatively rare tumors with small sample size, we analyzed the mRNA expression of ACSS2 using the European Genome- phenome Archive (EGA) database. The results surfaced that ACSS2 expression was higher in pNENs tumor tissues than in paraneoplastic tissues (Fig. 1E). To better explore the role of ACSS2 in pNENs, the expression of ACSS2 in pNENs cell lines was found to be higher than that in normal human pancreatic cell lines by qRT-PCR and WB (Fig. 1F, G), and immunofluorescence also showed the location of ACSS2 in each cell line (Fig. 1H).

### Overexpression of ACSS2 promoted proliferation, migration and invasion of pNENs cells

**Fig. 2:**
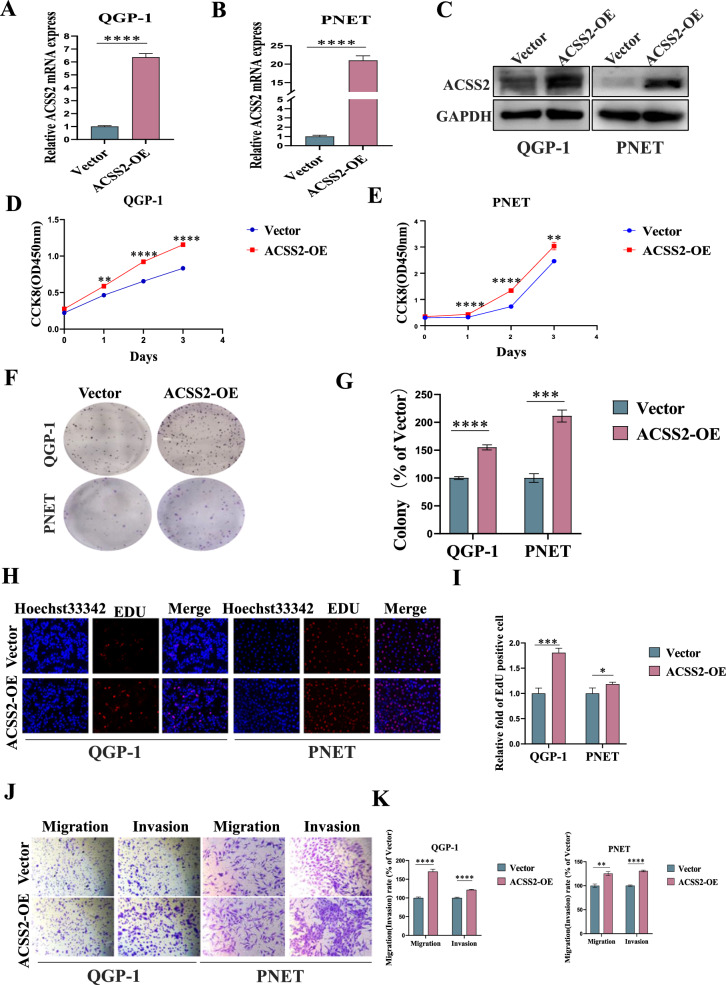
Overexpression of ACSS2 promoted pNENs cells viability. **A**–**C** pNENs cell lines stably overexpression of ACSS2 was constructed and assayed by qRT-PCR and WB. **D**, **E** CCK-8 assay showed that overexpression of ACSS2 promoted pNENs cell proliferation rate. **F**, **G** The colony formation indicated that overexpression of ACSS2 promoted pNENs cells proliferation. **H**, **I** Overexpression of ACSS2 significantly stimulated DNA synthesis. **J**, **K** Overexpression of ACSS2 successfully promoted the migration and invasion of pNENs cells. **p < 0.01, ***p < 0.001, ****p < 0.0001

To investigate the role of ACSS2 in pNENs, we established QGP-1, PNET cell lines stably overexpressing of ACSS2 and examined their transfection efficiency by qRT-PCR and WB (Fig. 2A–C). CCK-8 assay and colony formation assay and EdU assay showed that overexpression of ACSS2 promoted the proliferation of pNENs cells (Fig. 2D–I). Transwell assay showed that overexpression of ACSS2 promoted the migration and invasion ability of pNENs cells (Fig. 2J, K). Overall, these results confirmed that overexpression of ACSS2 facilitated pNENs cell proliferation, migration and invasion.

### Knockdown of ACSS2 and ACSS2i suppressed proliferation, migration and invasion of pNENs cells

**Fig. 3:**
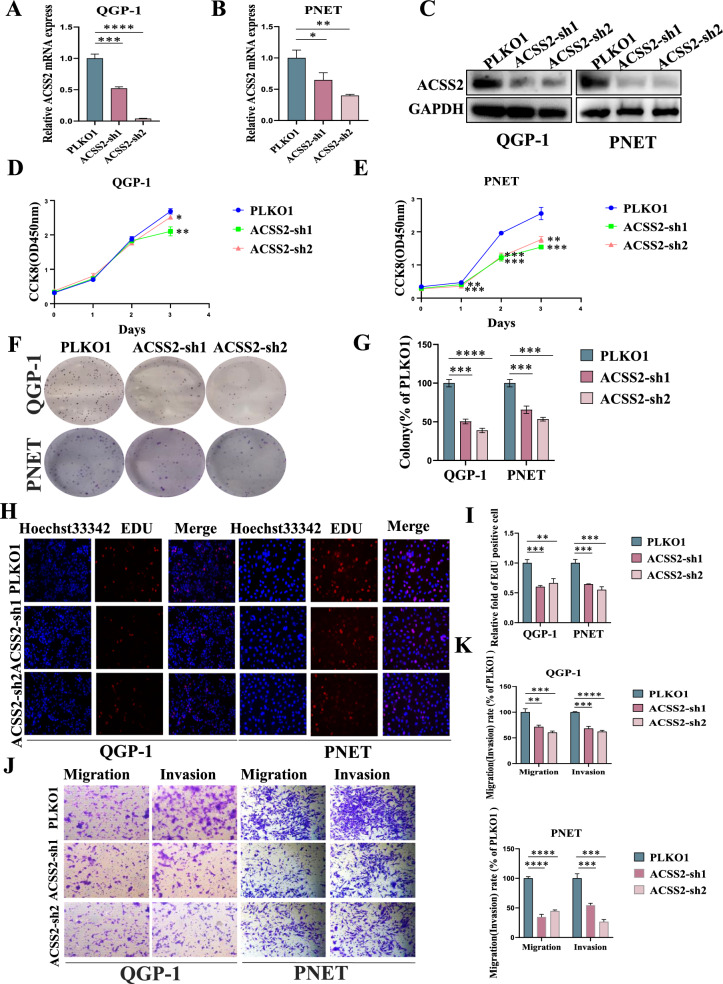
Knockdown of ACSS2 suppressed pNENs cells viability. **A**–**C** pNENs cell lines stably knockdown of ACSS2 was constructed and assayed by qRT-PCR and WB. **D**, **E** CCK-8 assay indicated that knockdown of ACSS2 inhibited pNENs cells proliferation. **F**, **G** The colony formation indicated that knockdown of ACSS2 suppressed pNENs cells proliferation. **H**, **I** Knockdown of ACSS2 significantly inhibited DNA synthesis. **J**, **K** Knockdown of ACSS2 successfully inhibited the migration and invasion of pNENs cells. *p < 0.05, **p < 0.01, ***p < 0.001, ****p < 0.0001

To further investigate the function of ACSS2 in pNENs, we constructed stably knockdown of ACSS2 QGP-1, PNET cell lines with lentivirus and confirmed the transfection efficiency by qRT-PCR and WB (Fig. 3A–C). CCK8 assay and colony formation assay showed that knockdown of ACSS2 inhibited the proliferation of pNENs cells (Fig. 3D–G). EdU assay also confirmed the inhibitory effect of knockdown of ACSS2 in pNENs cells (Fig. 3H, I). Transwell assay demonstrated that knockdown of ACSS2 suppressed the migration and invasion of pNENs cells (Fig. 3J, K). Further, we treated pNENs cells with ACSS2i and detected the IC50 of the action of ACSS2i in pNENs cells (Additional file 1: Fig. S4A, B). pNENs cells QGP-1 and PNET were treated separately according to the calculated IC50, and it was found that after ACSS2i treatment, the expression of ACSS2 was reduced (Additional file 1: Fig. S4C–E). Meanwhile, ACSS2i inhibited the proliferation, migration and invasion of pNENs cells (Additional file 1: Fig. S4F–M). In summary, the above results indicated that knockdown of ACSS2 and ACSS2i treatment significantly inhibited the proliferation, migration and invasion of pNENs cells.

### ACSS2 regulated lipid metabolism reprogramming in pNENs cells

**Fig. 4:**
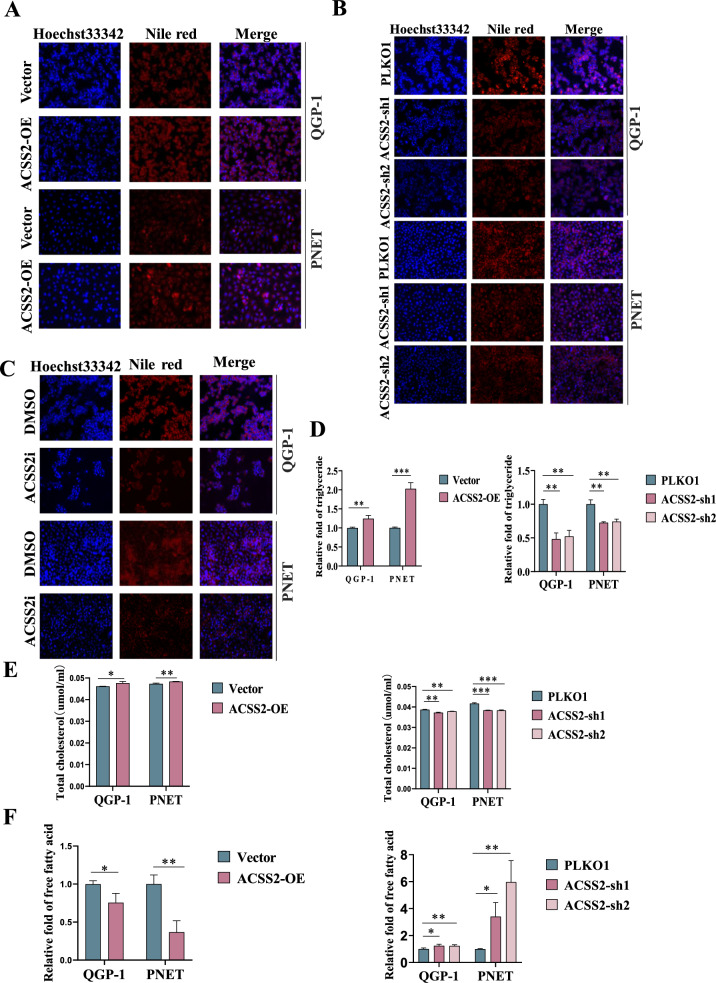
ACSS2 regulated lipid metabolism reprogramming in pNENs cells. **A** Increased lipid droplets after overexpression of ACSS2. **B**, **C** Knockdown of ACSS2 and ACSS2i treatment reduced lipid droplets. **D**, **E** Overexpression of ACSS2 increased triglyceride and total cholesterol, knockdown of ACSS2 reduced triglyceride and total cholesterol. **F** Overexpression of ACSS2 reduced free fatty acid and knockdown of ACSS2 increased free fatty acid. *p < 0.05, **p < 0.01, ***p < 0.001

ACSS2 is a lipid metabolism-related gene, so we performed metabolomics analysis on pNENs cells. We found that ACSS2 can regulate lipid metabolism reprogramming in pNENs cells (Additional file 1: Fig. S5A, B). Immediately we performed relevant in vitro experiments on lipid metabolism and found that lipid droplets increased with overexpression of ACSS2 and decreased with knockdown of ACSS2 and ACSS2i treatment in pNENs cells by Nile Red staining assay (Fig. 4A–C). We also examined the triglyceride and total cholesterol contents of different pNENs cells, and found that the triglyceride and total cholesterol contents were increased after overexpression of ACSS2, while the triglyceride and total cholesterol contents were decreased after knockdown of ACSS2 (Fig. 4D–E). Finally, it was found that after overexpression of ACSS2, the free fatty acid content of pNENs cells was reduced, whereas after knockdown of ACSS2, the free fatty acid content was increased in pNENs cells, which may be caused by ACSS2 promoting lipid metabolism and decreasing free fatty acid (Fig. 4F). Overall, ACSS2 can modulate lipid metabolism reprogramming in pNENs cells.

### ACSS2 regulated the PI3K/AKT/mTOR pathway and then enhanced lipid metabolism reprogramming in pNENs

**Fig. 5:**
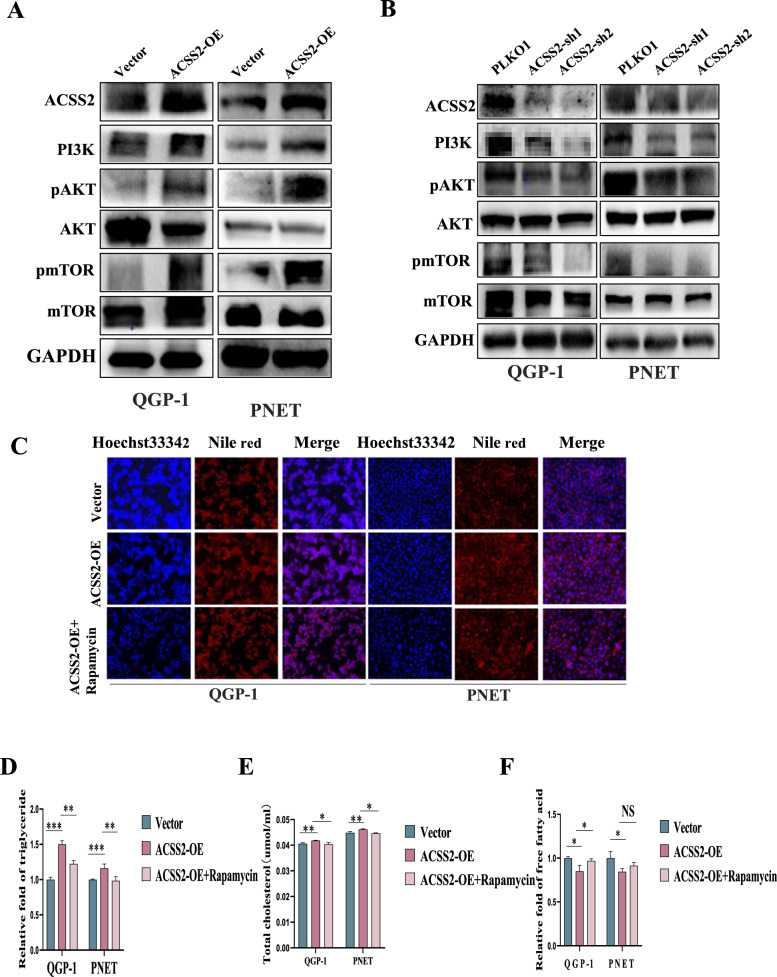
ACSS2 regulated the PI3K/AKT/mTOR pathway and then regulated lipid metabolism reprogramming. **A** Overexpression of ACSS2 activated the PI3K/AKT/mTOR pathway. **B** Knockdown of ACSS2 suppressed the PI3K/AKT/mTOR pathway. **C** Lipid droplets reduced after rapamycin-treated. **D**, **E** Triglycerides and total cholesterol reduced after rapamycin-treated (**F**). Free fatty acids increased after rapamycin-treated. *p < 0.05, **p < 0.01, ***p < 0.001

RNA- seq assays were used to study the molecular mechanism of ACSS2 in pNENs, and KEGG pathway analysis demonstrated that ACSS2 was connected to the PI3K/AKT/mTOR pathway (Additional file 1: Fig. S5C, D). Then we confirmed that ACSS2 could activate the PI3K/AKT/mTOR pathway by regulating related biomarkers PI3K、pAKT、pmTOR. The results revealed that the PI3K/AKT/mTOR pathway was activated when ACSS2 was overexpressed (Fig. 5A), and a reverse tendency showed when ACSS2 was knocked down (Fig. 5B). To further verify whether ACSS2 inhibited lipid metabolism reprogramming through the PI3K/AKT/mTOR pathway, we chose to treat overexpression of ACSS2 pNENs cells with rapamycin, an inhibitor of mTOR, and found that compared with overexpression of ACSS2 pNENs cells, rapamycin treatment pNENs cells showed a decrease in lipid droplets (Fig. 5C), as well as a decrease in triglyceride and total cholesterol (Fig. 5D, E), and an increase in free fatty acids (Fig. 5F). These results clearly indicated that ACSS2 regulated the PI3K/AKT/mTOR pathway and then enhanced lipid metabolism reprogramming in pNENs.

### HMGCS1 reverses the oncogenic effects of ACSS2 in vitro and in vivo

**Fig. 6:**
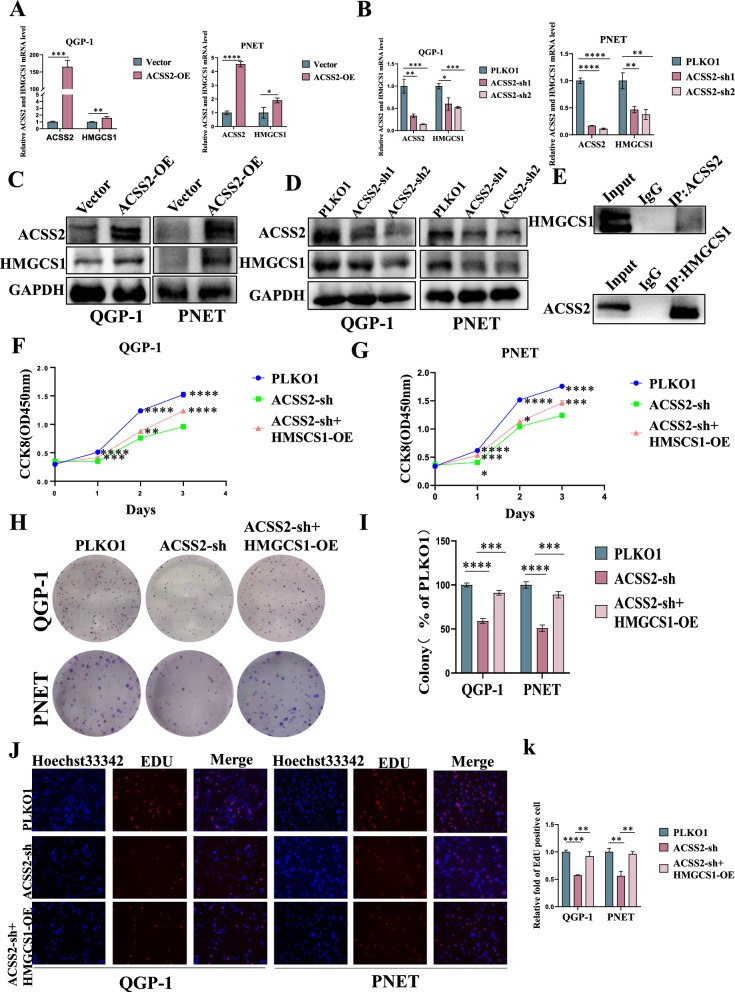
HMGCS1 reversed the oncogenic effects of ACSS2 in vitro. **A**–**D** Overexpression of ACSS2 upregulated the expression of HMGCS1 and knockdown of ACSS2 downregulated the expression of HMGCS1 by qRT-PCR and WB. **E** Co-IP demonstrated ACSS2 and HMGCS1 interaction. **F**, **G **CCK-8 assay indicated that overexpression of HMGCS1 reversed the tumor-suppressive effect of ACSS2 knocked down. **H**, **I** The colony formation suggested that overexpression of HMGCS1 reversed the tumor-suppressive effect of ACSS2 knocked down. **J**, **K** EdU assay indicated that overexpression of HMGCS1 reversed the DNA synthesis suppressed effect of ACSS2 knocked down. *p < 0.05, **p < 0.01, ***p < 0.001, ****p < 0.0001

**Fig. 7:**
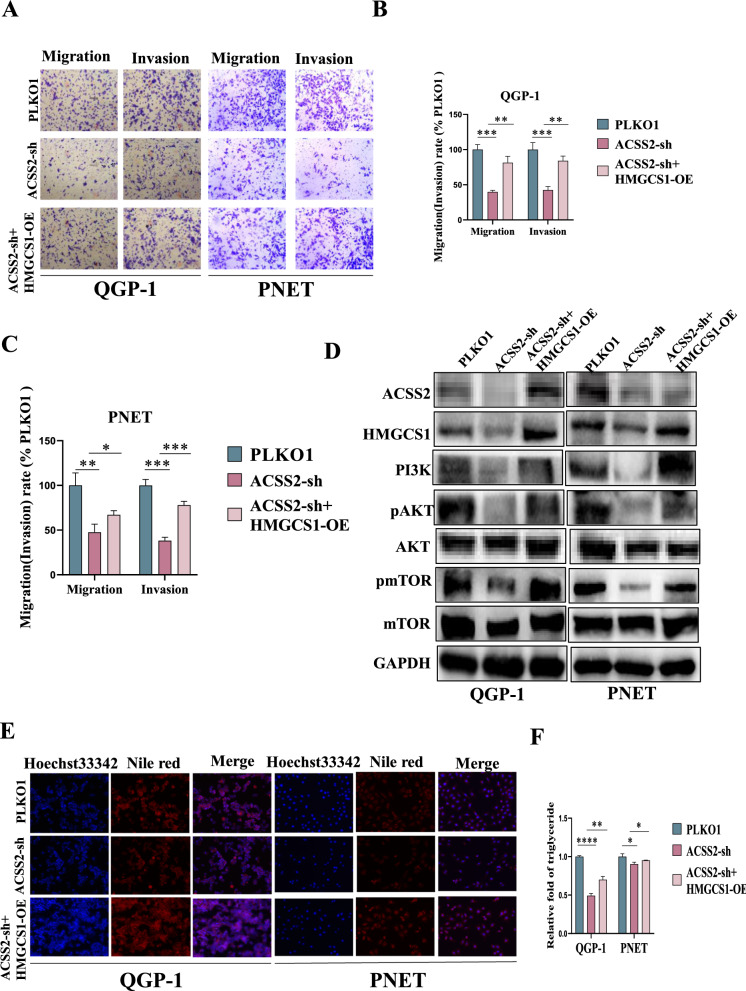
HMGCS1 reversed the oncogenic effects of ACSS2 and regulated lipid metabolism. **A**–**C** Overexpression of HMGCS1 reversed the inhibitory effect of ACSS2 knocked down on pNENs cell migration and invasion. **D** Overexpression of HMGCS1 reversed the inhibitory effect of ACSS2 knocked down on PI3K/AKT/mTOR pathway. **E** Overexpression of HMGCS1 in ACSS2 knocked down pNENs cells increased the lipid droplets. **F** Overexpression of HMGCS1 in ACSS2 knocked down pNENs cells increased the triglyceride. *p < 0.05, **p < 0.01, ***p < 0.001, ****p < 0.0001

**Fig. 8:**
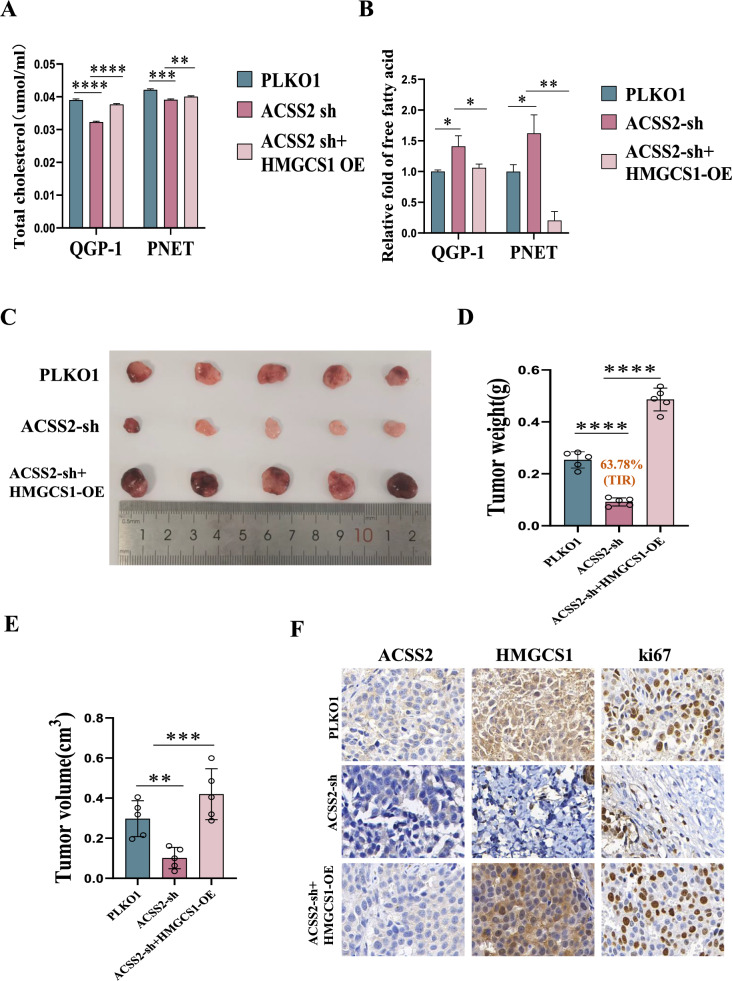
HMGCS1 regulated lipid metabolism and reversed the oncogenic effects of ACSS2 in vivo. **A** Overexpression of HMGCS1 in ACSS2 knocked down pNENs cells increased the cholesterol. **B** Overexpression of HMGCS1 in ACSS2 knocked down pNENs cells reduced the free fatty acids. **C**–**E** Tumor weight and volume were smaller in the ACSS2 knocked down group and further larger in the overexpression of HMGCS1 of ACSS2 knocked down group, The average TIR of ACSS2-sh group was 63.78%. **F** Knockdown of ACSS2 and overexpression of HMGCS1 of ACSS2 Knocked down successfully, Knockdown of ACSS2 inhibited the expression of proliferative index Ki67, while overexpression of HMGCS1 promotes the expression of proliferative index Ki67. *p < 0.05,**p < 0.01, ***p < 0.001, ****p < 0.0001

To further explore through which relevant lipid metabolism molecules ACSS2 facilitated the progression of pNENs, we performed IP experiments and sequencing results revealed that HMGCS1 is a lipid metabolism-related protein that interacted with ACSS2 in pNENs cells. To verify the relationship between HMGCS1 and ACSS2 in pNENs cells, qRT-PCR and WB indicated that overexpression of ACSS2 in pNENs cells was followed by elevated expression of HMGCS1, and at the same time, knockdown of ACSS2 in pNENs cells was followed by decreased expression of HMGCS1 (Fig. 6A–D). Similarly, the results of Co-IP assay showed that the HMGCS1 protein interacted with the ACSS2 protein (Fig. 6E). CCK8 assay, colony formation assay, EdU assay and transwell assay showed that overexpression of HMGCS1 in ACSS2 knocked down pNENs cells promotes the proliferation and migratory invasion of pNENs cells (Figs 6F–K, 7A–C). Overexpression of HMGCS1 in ACSS2 knocked down pNENs cells was further found to activate the PI3K/AKT/mTOR pathway by WB experiments (Fig. 7D). We also explored the potential effects of HMGCS1 on lipid metabolism and found that overexpression of HMGCS1 in ACSS2 knocked down pNENs cells resulted in increased lipid droplets, as well as an increase in triglycerides and total cholesterol, and fewer free fatty acids (Figs. 7E, F, 8A, B). To further evaluate the effect of ACSS2 on tumors in vivo, we constructed a nude mouse xenograft model. Nude mice were injected subcutaneously with 5 × 10^6^ QGP-1 cells in the axilla, and three groups of mice were injected: (1)wild-type QGP-1 cells; (2) knockdown of ACSS2 QGP-1 cells; (3) overexpression of HMGCS1 in ACSS2 knocked down QGP-1 cells, and the nude mice were euthanized after 5 weeks. Tumor weight and volume were smaller in the knockdown of ACSS2 group compared to the wild-type group, and further larger in the overexpression of HMGCS1 in ACSS2 knocked down group compared to the knockdown of ACSS2 group. Meanwhile, the average TIR of ACSS2 Knocked down group reached 63.78% (Fig. 8C–E). Immunohistochemical determined that ACSS2 was successfully knocked down in the knockdown of ACSS2 group and HMGCS1 was successfully overexpressed in the overexpression of HMGCS1 in ACSS2 knocked down group. Knockdown of ACSS2 inhibited the expression of proliferative index Ki67, while overexpression of HMGCS1 promoted the expression of proliferative index Ki67 (Fig. 8F). In conclusion, these results established that ACSS2 affects the PI3K/AKT/mTOR pathway through the downstream molecule HMGCS1 thereby promoting the progression of pNENs.

## Discussion

Hypoxia is a common feature of tumors with poor clinical prognosis that have aggressive features such as increased metabolic and migratory potential. Previous studies have shown that lipid metabolism is drastically altered under hypoxic conditions [12]. Hypoxia has been shown to upregulate the lipid metabolism-related gene ACSS2 in prostate and breast cancers [13]. When oxygen is scarce, cells adapt to the lack of oxygen by reprogramming the expression of some genes involved in energy metabolism. The role of HIF-1 in the activation of protein-coding genes involved in carbohydrate metabolism has long been established [14, 15]. The role of hypoxia in pNENs has not yet been revealed. Therefore, our study first revealed the role played by hypoxia in pNENs, and it was found that hypoxia could promote the progression of pNENs. In order to explore the role played by hypoxia on lipid metabolism in pNENs, we found that hypoxia could up-regulate lipid metabolism-related gene ACSS2 and both of them were regulated by each other at the transcriptional level.

Studies over the past two decades have clearly established that altered lipid metabolism is an important metabolic phenotype in cancer cells. Therefore, blocking the lipid supply to cancer cells would have a dramatic impact on cancer cell bioenergetics, membrane biosynthesis and intracellular signaling processes [9]. ACSS2 is a key factor in lipid metabolism that functions differently in different tumors. ACSS2 is expressed in many tumors, localized in the mammalian cytoplasm and nucleus, and is responsible for catalyzing the generation of acetyl coenzyme A, an intermediate product of metabolism from acetate [16]. Under metabolic stress conditions, ACSS2 promotes the growth of cancer cells [13], and ACSS2 deficiency inhibits the growth of tumor cells and tumor formation in mice [17]. Moreover, ACSS2 is upregulated in renal cell carcinoma and promotes migration and invasion of renal cancer cells [18]. Our research indicated that ACSS2 was highly expressed in pNENs, and overexpression of ACSS2 promoted the proliferation, migration and invasion of pNENs, while knockdown of ACSS2 and ACSS2i treatment inhibited the proliferation of pNENs after intervention. We also found that ACSS2 regulated lipid metabolism. Therefore, ACSS2 may promote the progression of pNENs by regulating lipid metabolism.

Our study found a protein molecule HMGCS1 interacts with ACSS2by IP assay. Moreover, WB and Co-IP analysis showed that ACSS2 interacted with HMGCS1 and that ACSS2 regulated the expression of HMGCS1. ACSS2 is the key enzyme for the synthesis of acetyl-CoA, which is the substrate for the acetoacetyl-CoA. HMGCS1 is the first key enzyme of the mevalonate pathway that converts acetoacetyl-CoA to HMG-CoA [19]. Previous studies have demonstrated that HMGCS1 expression is associated with malignant progression in colon cancer, gastric cancer, hepatocellular carcinoma, cervical cancer and cutaneous squamous cell carcinomas [20]. Recent studies demonstrated that HMGCS1 is a potential metabolic target for the treatment of cancer [21]. HMGCS1 expression is upregulated in many tumors and can promote tumor progression through both metabolic and non-metabolic pathways [22]. Our study indicated that overexpression of ACSS2 in pNENs promoted HMGCS1 expression, and knockdown of ACSS2 gave the opposite result. More recently, we found in vivo and in vitro that overexpression of HMGCS1 reversed the oncogenic effect of ACSS2 knockdown on pNENs. Meanwhile, overexpression of HMGCS1 can reverse the regulation of lipid metabolism by ACSS2 knockdown. Our results revealed that HMGCS1 may alter the progression of pNENs through metabolic pathway. Currently, there are no reports on the roles of ACSS2 and HMGCS1 in pNENs, and only early clinical trials of ACSS2i in tumors are underway. In our study, we revealed that the downstream molecule of ACSS2 regulating the progression of pNENs is HMGCS1, and ACCS2 affects the proliferation of pNENs by interfering with the expression of HMGCS1. Therefore, our findings provide a new therapeutic targets for pNENs, and intervention with ACSS2 or HMGCS1 may become a new potential therapeutic target for pNENs.

In addition, we performed RNA-seq analysis to investigate knockdown of ACSS2 for relative biological difference analysis. The results revealed that ACSS2 may be associated with the PI3K/AKT/mTOR pathway, which was further verified by WB detection of the expression of biomarker proteins(such as PI3K、pAKT and pmTOR) related to the PI3K/AKT/mTOR pathway. The PI3K/AKT/mTOR pathway is one of the intracellular signaling pathways, which has an impact on cell metabolism, proliferation and survival [23]. Numerous studies have shown it to be associated with a growing number of tumors, including colorectal, breast, prostate and ovarian cancers [24]. Abnormal activation of the PI3K/AKT/mTOR signaling pathway plays an important role in tumor cell proliferation, survival, angiogenesis, invasion and migration [25]. The PI3K/AKT/mTOR signaling pathway is an important coordinator of pNET cell growth, proliferation, angiogenesis and metabolism. Based on the efficacy shown in the phase III randomized Radiant-3 trial, the mTOR inhibitor everolimus has become a standard treatment option for advanced pNETs [26]. Our study expressed that overexpression of ACSS2 activated the PI3K/AKT/mTOR pathway, whereas knockdown of ACSS2 inhibited the PI3K/AKT/mTOR pathway. Meanwhile, ACSS2 regulation of lipid metabolism can be inhibited by the mTOR inhibitor rapamycin. Moreover, HMGCS1 can reverse the inhibitory effect of knockdown of ACSS2 on the PI3K/AKT/mTOR pathway.

**Fig. 9:**
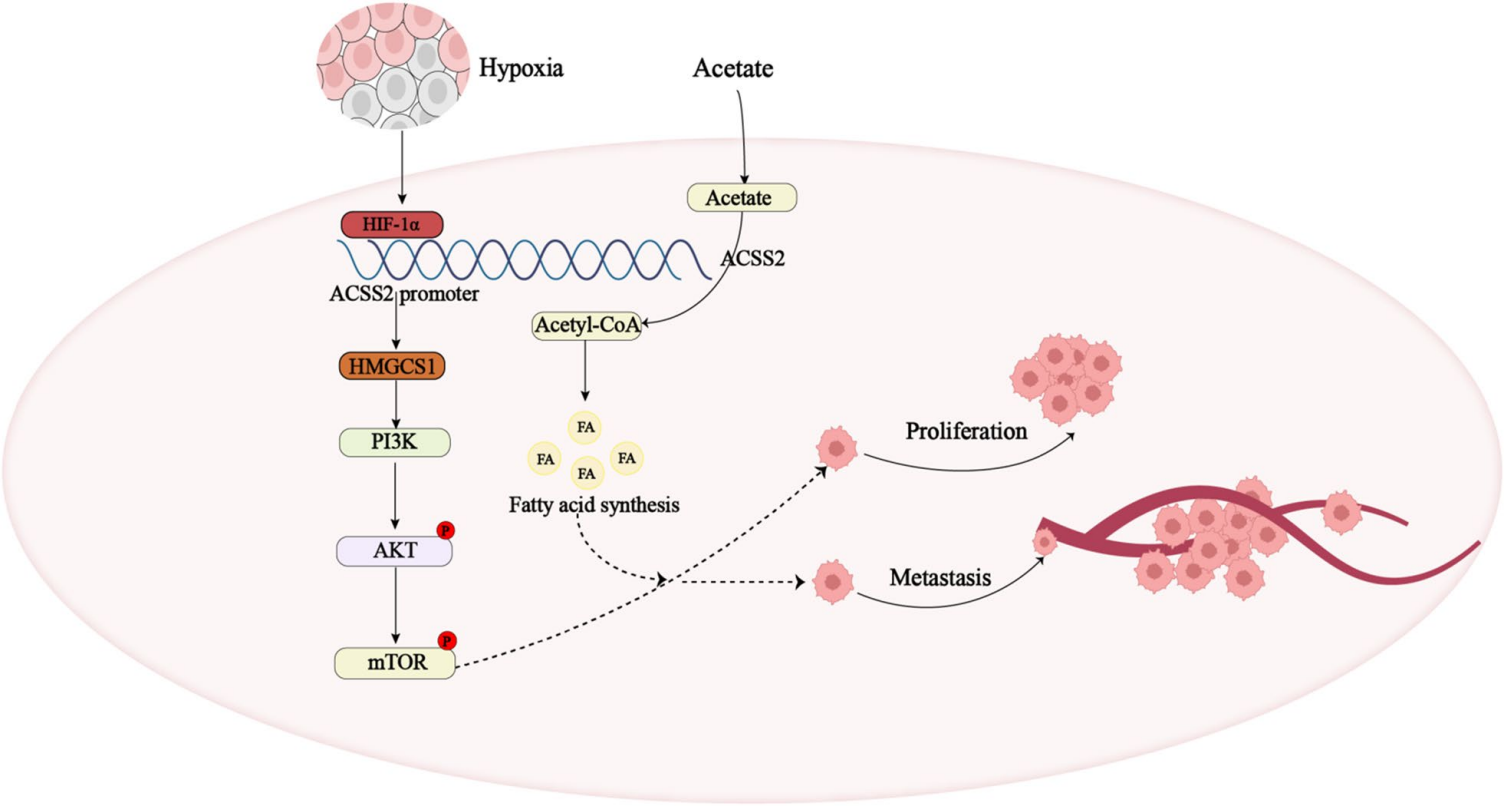
Schematic diagram showing that hypoxia can upregulate a lipid metabolism-related gene ACSS2 in pNENs, and the upregulated ACSS2 gene in pNENs can enhance lipid metabolism reprogramming, which further regulated the expression of HMGCS1 to affect the PI3K/AKT/mTOR pathway to promote the progression of pNENs. In addition, we found that HMGCS1 can reverse the regulation of lipid metabolism exerted by ACSS2, as well as the pro-tumorigenic role of ACSS2 in pNENs, which has also been detected in vitro and in vivo

In conclusion, our findings indicated that hypoxia can upregulate a lipid metabolism-related gene ACSS2 in pNENs, and the upregulated ACSS2 gene in pNENs can enhance lipid metabolism reprogramming, which further regulated the expression of HMGCS1 to affect the PI3K/AKT/mTOR pathway to promote the progression of pNENs. In addition, we found that HMGCS1 can reverse the regulation of lipid metabolism exerted by ACSS2, as well as the pro-tumorigenic role of ACSS2 in pNENs, which has also been detected in vitro and in vivo (Fig. 9). Our study not only identified a potential early diagnostic biomarker for pNENs but also provided a potential therapeutic target of pNENs.

### Supplementary Information

**Additional file 1: Figure S1.** Hypoxia promoted pNENs cells viability. (A, B)CCK-8 assay indicated that hypoxia can promote pNENs cells proliferation rate. (C, D)Hypoxia facilitated pNENs cells colony formation ability. (E, F) Hypoxia significantly promoted DNA synthesis. (G, H)Hypoxia successfully promoted the migration and invasion ability of pNENs cells. *p < 0.05, **p < 0.01, ***p < 0.001, ****p < 0.0001. **Figure S2.** Overexpression of HIF-1a promoted pNENs cells viability. (A, B, C) pNENs cell lines stably overexpression of HIF-1a was constructed and assayed by qRT-PCR and WB. (D, E)CCK-8 assay showed that overexpression of HIF-1a promoted pNENs cell proliferation rate. (F, G)The colony formation indicated that overexpression of HIF-1a promoted pNENs cells proliferation. (H, I)Overexpression of HIF-1a significantly stimulated DNA synthesis. (J, K)Overexpression of HIF-1a successfully promoted the migration and invasion of pNENs cells. **p < 0.01, ***p < 0.001, ****p < 0.0001. **Figure S3.** Knockdown of HIF-1a suppressed pNENs cells viability. (A, B, C) pNENs cell lines stably knockdown of HIF-1a was constructed and assayed by qRT-PCR and WB. (D, E)CCK-8 assay indicated that knockdown of HIF-1a inhibited pNENs cells proliferation. (F, G) The colony formation indicated that knockdown of HIF-1a suppressed pNENs cells proliferation. (H, I) Knockdown of HIF-1a significantly inhibited DNA synthesis. (J, K) Knockdown of HIF-1a successfully inhibited the migration and invasion of pNENs cells. *p < 0.05, **p < 0.01, ***p < 0.001, ****p < 0.0001. **Figure S4.** ACSS2i suppressed pNENs cells viability. (A, B) The IC50 of ACSS2i action in pNENs cell. (C, D, E) ACSS2i treatment pNENs downregulates ACSS2 expression in pNENs cells and assayed by qRT-PCR and WB. (F, G) CCK-8 assay indicated that ACSS2i inhibited pNENs cells proliferation. (H, I) The colony formation indicated that ACSS2i suppressed pNENs cells proliferation. (J, K) ACSS2i significantly inhibited DNA synthesis. (L, M) ACSS2i successfully inhibited the migration and invasion of pNENs cells. **p < 0.01, ***p < 0.001, ****p < 0.0001. **Figure S5.** Metabolomics analysis and RNA-seq assays regulated lipid metabolism and PI3K/AKT/mTOR pathyway. (A, B) Metabolomics analysis suggested that ACSS2 regulated lipid metabolism in pNENs. (C, D) RNA-seq assays indicated that ACSS2 regulated PI3K/AKT/mTOR pathway.

**Additional file 2: Table S1.** Primers and shRNA used in the study. **Table S2. **Antibodies used in the study.

## Data Availability

All data generated or analyzed during this study are included in this published article.

## References

[1] Dasari A, Shen C, Halperin D (2017). Trends in the incidence, prevalence, and survival outcomes in patients with neuroendocrine tumors in the United States. JAMA Oncol..

[2] Scott AT, Howe JR (2019). Evaluation and management of neuroendocrine tumors of the pancreas. Surg Clin North Am.

[3] Hallet J, Law CH, Cukier M, Saskin R, Liu N, Singh S (2015). Exploring the rising incidence of neuro-endocrine tumors: a population-based analysis of epidemiology, metastatic presentation, and outcomes. Cancer.

[4] Ma ZY, Gong YF, Zhuang HK (2020). Pancreatic neuroendocrine tumors: a review of serum biomarkers, staging, and management. World J Gastroenterol.

[5] Chabot J (2016). Editorial: pancreatic neuroendocrine tumors: primum non nocere. Surgery.

[6] Fang JM, Shi JA (2019). Clinicopathologic and molecular update of pancreatic neuroendocrine neoplasms with a focus on the new world health organization classification. Arch Pathol Lab Med..

[7] Li Y, Zhao L, Li XF (2021). Hypoxia and the tumor microenvironment. Technol Cancer Res Treat.

[8] Mylonis I, Simos G, Paraskeva E (2019). Hypoxia-inducible factors and the regulation of lipid metabolism. Cells.

[9] Röhrig F, Schulze A (2016). The multifaceted roles of fatty acid synthesis in cancer. Nat Rev Cancer.

[10] Röhrig F, Schulze A (2016). The multifaceted roles of fatty acid synthesis in cancer. Nat Rev Cancer..

[11] Lu F, Ye M, Hu C (2023). FABP5 regulates lipid metabolism to facilitate pancreatic neuroendocrine neoplasms progression via FASN mediated Wnt/β-catenin pathway. Cancer Sci.

[12] Ackerman D, Simon MC (2014). Hypoxia, lipids, and cancer: surviving the harsh tumor microenvironment. Trends Cell Biol.

[13] Schug ZT, Peck B, Jones DT (2015). Acetyl-CoA synthetase 2 promotes acetate utilization and maintains cancer cell growth under metabolic stress. Cancer Cell.

[14] Samanta D, Semenza GL (2018). Metabolic adaptation of cancer and immune cells mediated by hypoxia-inducible factors. Biochim Biophys Acta Rev Cancer.

[15] Xie H, Simon MC (2017). Oxygen availability and metabolic reprogramming in cancer. J Biol Chem.

[16] Li X, Yu W, Qian X (2017). Nucleus-translocated ACSS2 promotes gene transcription for lysosomal biogenesis and autophagy. Mol Cell.

[17] Comerford SA, Huang Z, Du X (2014). Acetate dependence of tumors. Cell.

[18] Yao L, Guo X, Gui Y (2018). Acetyl-CoA Synthetase 2 promotes cell migration and invasion of renal cell carcinoma by upregulating lysosomal-associated membrane protein 1 expression. Cell Physiol Biochem.

[19] Chen Y, Li M, Yang Y, Lu Y, Li X (2022). Antidiabetic drug metformin suppresses tumorigenesis through inhibition of mevalonate pathway enzyme HMGCS1. J Biol Chem.

[20] Xiao MY, Li FF, Xie P (2023). Gypenosides suppress hepatocellular carcinoma cells by blocking cholesterol biosynthesis through inhibition of MVA pathway enzyme HMGCS1. Chem Biol Interact..

[21] Xu H, Zhou S, Tang Q, Xia H, Bi F (2020). Cholesterol metabolism: new functions and therapeutic approaches in cancer. Biochim Biophys Acta Rev Cancer.

[22] Wang IH, Huang TT, Chen JL (2020). Mevalonate pathway enzyme HMGCS1 contributes to gastric cancer progression. Cancers (Basel)..

[23] Ediriweera MK, Tennekoon KH, Samarakoon SR (2019). Role of the PI3K/AKT/mTOR signaling pathway in ovarian cancer: biological and therapeutic significance. Semin Cancer Biol.

[24] Akbarzadeh M, Mihanfar A, Akbarzadeh S, Yousefi B, Majidinia M (2021). Crosstalk between miRNA and PI3K/AKT/mTOR signaling pathway in cancer. Life Sci.

[25] Liu R, Chen Y, Liu G (2020). PI3K/AKT pathway as a key link modulates the multidrug resistance of cancers. Cell Death Dis.

[26] Vernieri C, Pusceddu S, Fucà G (2019). Impact of systemic and tumor lipid metabolism on everolimus efficacy in advanced pancreatic neuroendocrine tumors (pNETs). Int J Cancer.

