# Supplementary material for: Hypoxia upregulating ACSS2 enhances lipid metabolism reprogramming through HMGCS1 mediated PI3K/AKT/mTOR pathway to promote the progression of pancreatic neuroendocrine neoplasms
Source: J Transl Med. 2024 Jan 23;22:93. doi: 10.1186/s12967-024-04870-z (PMC10804556; doi:10.1186/s12967-024-04870-z)
Supplement: Supplementary file 1 — Additional file 1: Figure S1. Hypoxia promoted pNENs cells viability. (A, B)CCK-8 assay indicated that hypoxia can promote pNENs cells proliferation rate. (C, D)Hypoxia facilitated pNENs cells colony formation ability. (E, F) Hypoxia significantly promoted DNA synthesis. (G, H)Hypoxia successfully promoted the migration and invasion ability of pNENs cells. *p < 0.05, **p < 0.01, ***p < 0.001, ****p < 0.0001. Figure S2. Overexpression of HIF-1a promoted pNENs cells viability. (A, B, C) pNENs cell lines stably overexpression of HIF-1a was constructed and assayed by qRT-PCR and WB. (D, E)CCK-8 assay showed that overexpression of HIF-1a promoted pNENs cell proliferation rate. (F, G)The colony formation indicated that overexpression of HIF-1a promoted pNENs cells proliferation. (H, I)Overexpression of HIF-1a significantly stimulated DNA synthesis. (J, K)Overexpression of HIF-1a successfully promoted the migration and invasion of pNENs cells. **p < 0.01, ***p < 0.001, ****p < 0.0001. Figure S3. Knockdown of HIF-1a suppressed pNENs cells viability. (A, B, C) pNENs cell lines stably knockdown of HIF-1a was constructed and assayed by qRT-PCR and WB. (D, E)CCK-8 assay indicated that knockdown of HIF-1a inhibited pNENs cells proliferation. (F, G) The colony formation indicated that knockdown of HIF-1a suppressed pNENs cells proliferation. (H, I) Knockdown of HIF-1a significantly inhibited DNA synthesis. (J, K) Knockdown of HIF-1a successfully inhibited the migration and invasion of pNENs cells. *p < 0.05, **p < 0.01, ***p < 0.001, ****p < 0.0001. Figure S4. ACSS2i suppressed pNENs cells viability. (A, B) The IC50 of ACSS2i action in pNENs cell. (C, D, E) ACSS2i treatment pNENs downregulates ACSS2 expression in pNENs cells and assayed by qRT-PCR and WB. (F, G) CCK-8 assay indicated that ACSS2i inhibited pNENs cells proliferation. (H, I) The colony formation indicated that ACSS2i suppressed pNENs cells proliferation. (J, K) ACSS2i significantly inhibited DNA synthesis. (L, M) AC [file 12967_2024_4870_MOESM1_ESM.pdf]

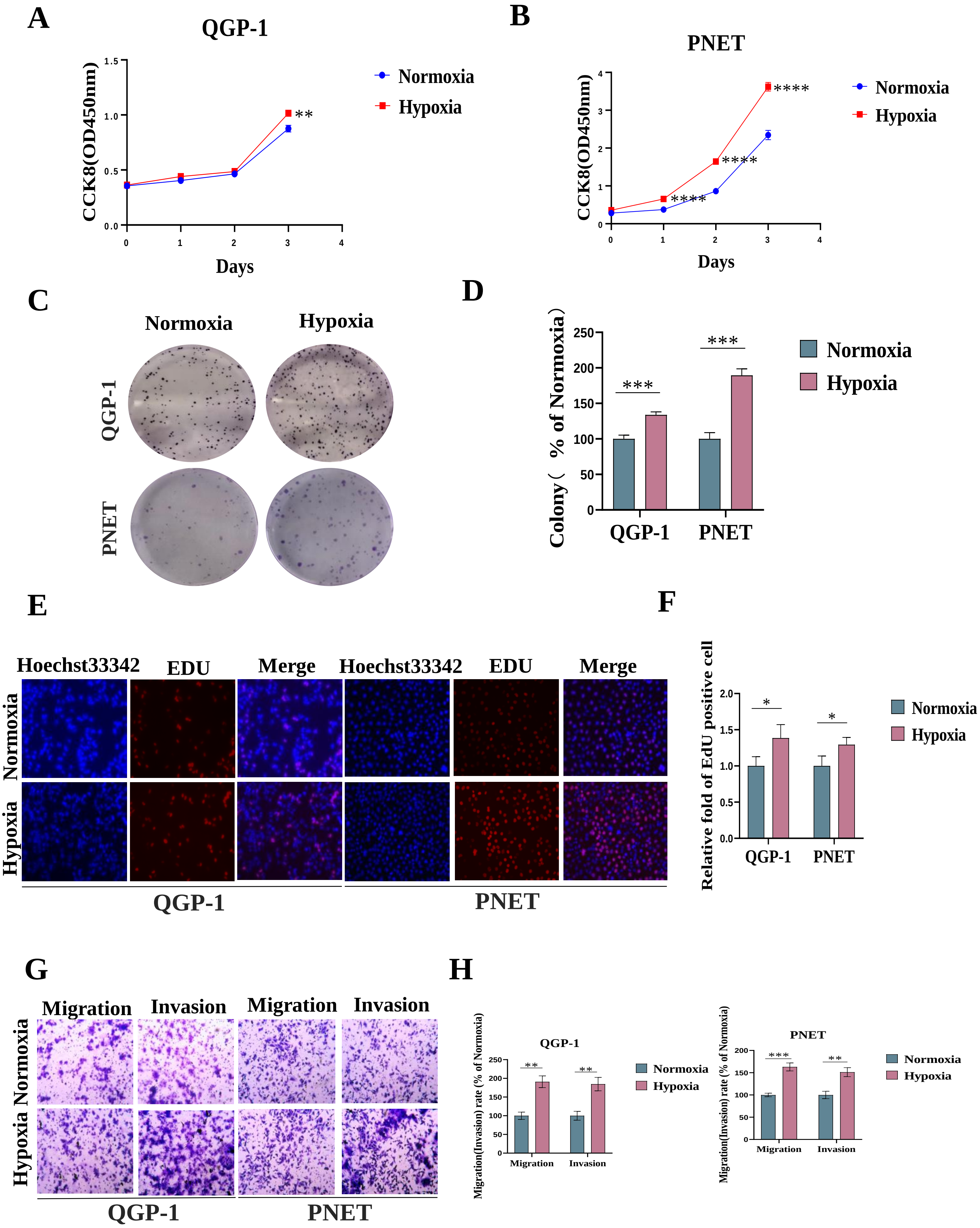
A
QGP-1
CCK8(OD450nm)
Days
Normoxia
Hypoxia
**
B
PNET
CCK8(OD450nm)
Days
Normoxia
Hypoxia
****
C
Normoxia
Hypoxia
QGP-1
PNET
D
Colony（ % of Normoxia）
QGP-1
PNET
Normoxia
Hypoxia
***
E
Hoechst33342
EDU
Merge
Normoxia
Hypoxia
QGP-1
PNET
F
Relative fold of EdU positive cell
QGP-1
PNET
Normoxia
Hypoxia
*
G
Migration
Invasion
Normoxia
Hypoxia
QGP-1
PNET
H
QGP-1
PNET
Migration(Invasion) rate (% of Normoxia)
Migration
Invasion
Normoxia
Hypoxia

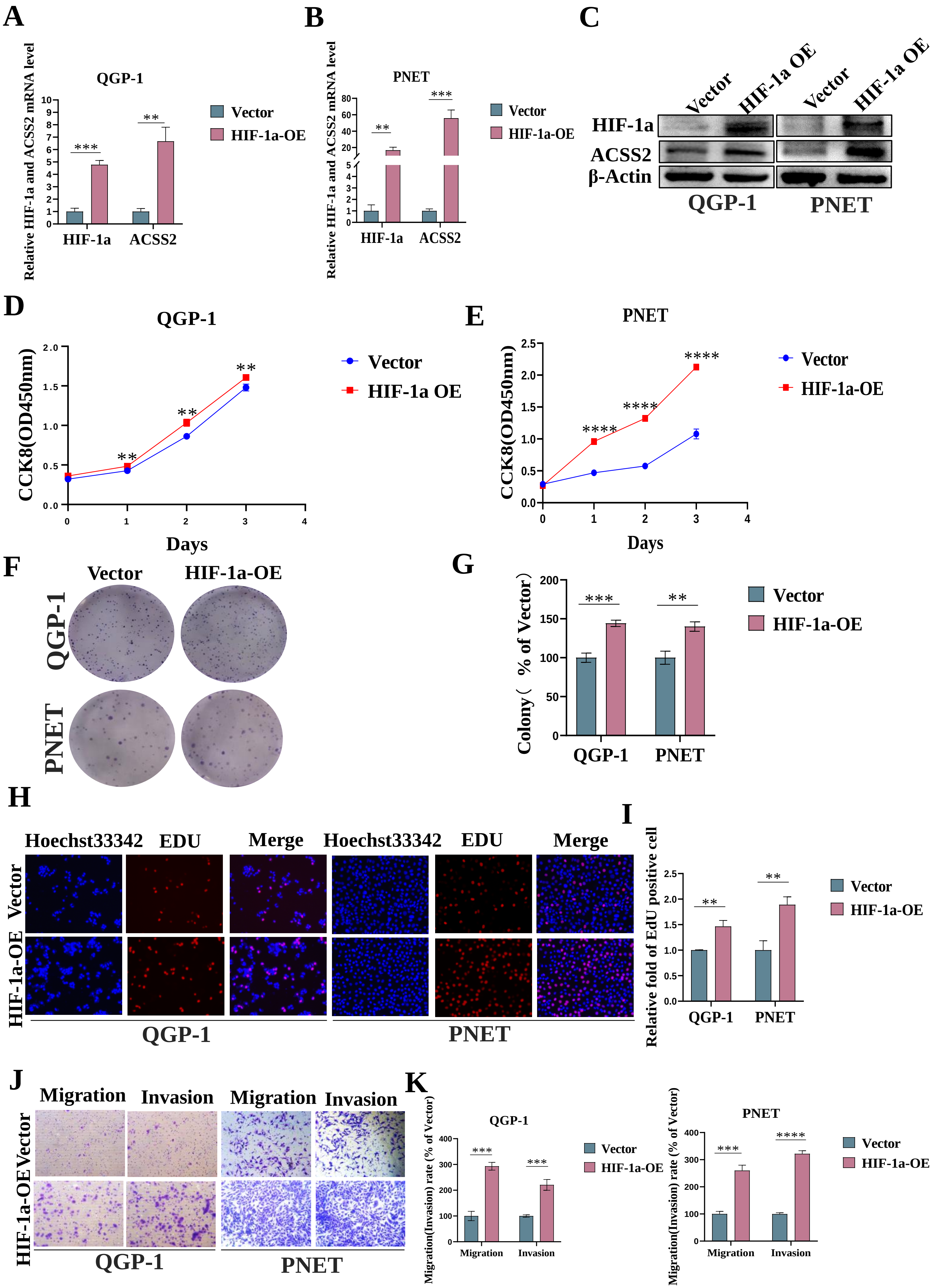

A
QGP-1
Relative HIF-1a and ACSS2 mRNA level
Vector
HIF-1a-OE
HIF-1a
ACSS2
B
PNET
Relative HIF-1a and ACSS2 mRNA level
Vector
HIF-1a-OE
HIF-1a
ACSS2
C
Vector
HIF-1a OE
Vector
HIF-1a OE
HIF-1a
ACSS2
β-Actin
QGP-1
PNET
D
QGP-1
CCK8(OD450nm)
Days
Vector
HIF-1a OE
E
PNET
CCK8(OD450nm)
Days
Vector
HIF-1a-OE
F
Vector
HIF-1a-OE
QGP-1
PNET
G
Colony（% of Vector）
QGP-1
PNET
Vector
HIF-1a-OE
H
Hoechst33342
EDU
Merge
Hoechst33342
EDU
Merge
Vector
HIF-1a-OE
QGP-1
PNET
I
Relative fold of EdU positive cell
QGP-1
PNET
Vector
HIF-1a-OE
J
Migration
Invasion
Migration
Invasion
Vector
HIF-1a-OE
QGP-1
PNET
K
QGP-1
Migration(Invasion) rate (% of Vector)
Migration
Invasion
Vector
HIF-1a-OE
PNET
Migration(Invasion) rate (% of Vector)
Migration
Invasion
Vector
HIF-1a-OE

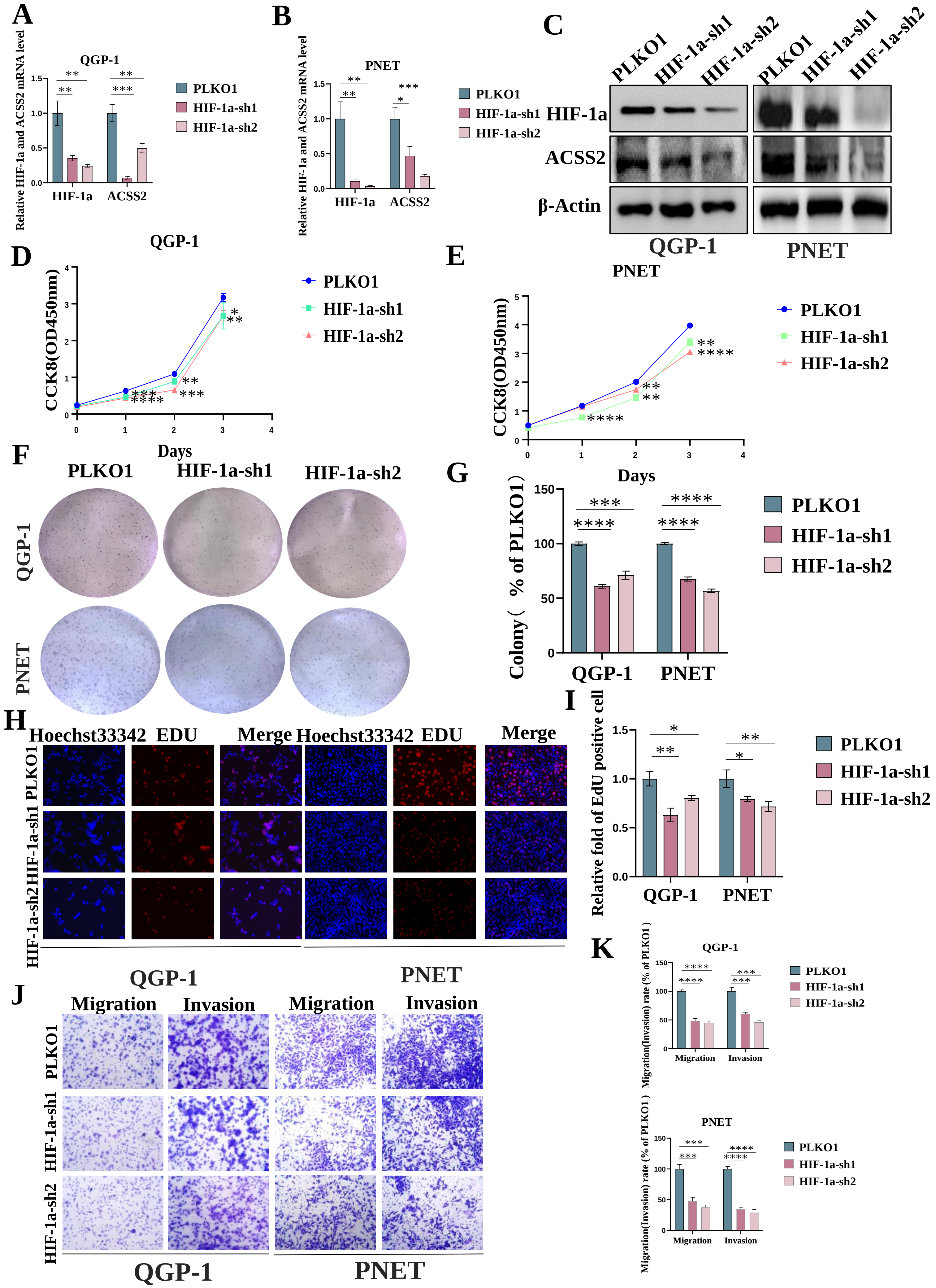

A
QGP-1
Relative HIF-1a and ACSS2 mRNA level
HIF-1a
ACSS2
PLKO1
HIF-1a-sh1
HIF-1a-sh2
B
PNET
Relative HIF-1a and ACSS2 mRNA level
C
PLKO1
HIF-1a-sh1
HIF-1a-sh2
HIF-1a
ACSS2
β-Actin
QGP-1
PNET
D
QGP-1
CCK8(OD450nm)
Days
E
PNET
CCK8(OD450nm)
Days
F
PLKO1
HIF-1a-sh1
HIF-1a-sh2
QGP-1
PNET
G
Colony（ % of PLKO1）
H
Hoechst33342
EDU
Merge
I
Relative fold of EdU positive cell
J
QGP-1
PNET
Migration
Invasion
K
Migration(Invasion) rate (% of PLKO1)

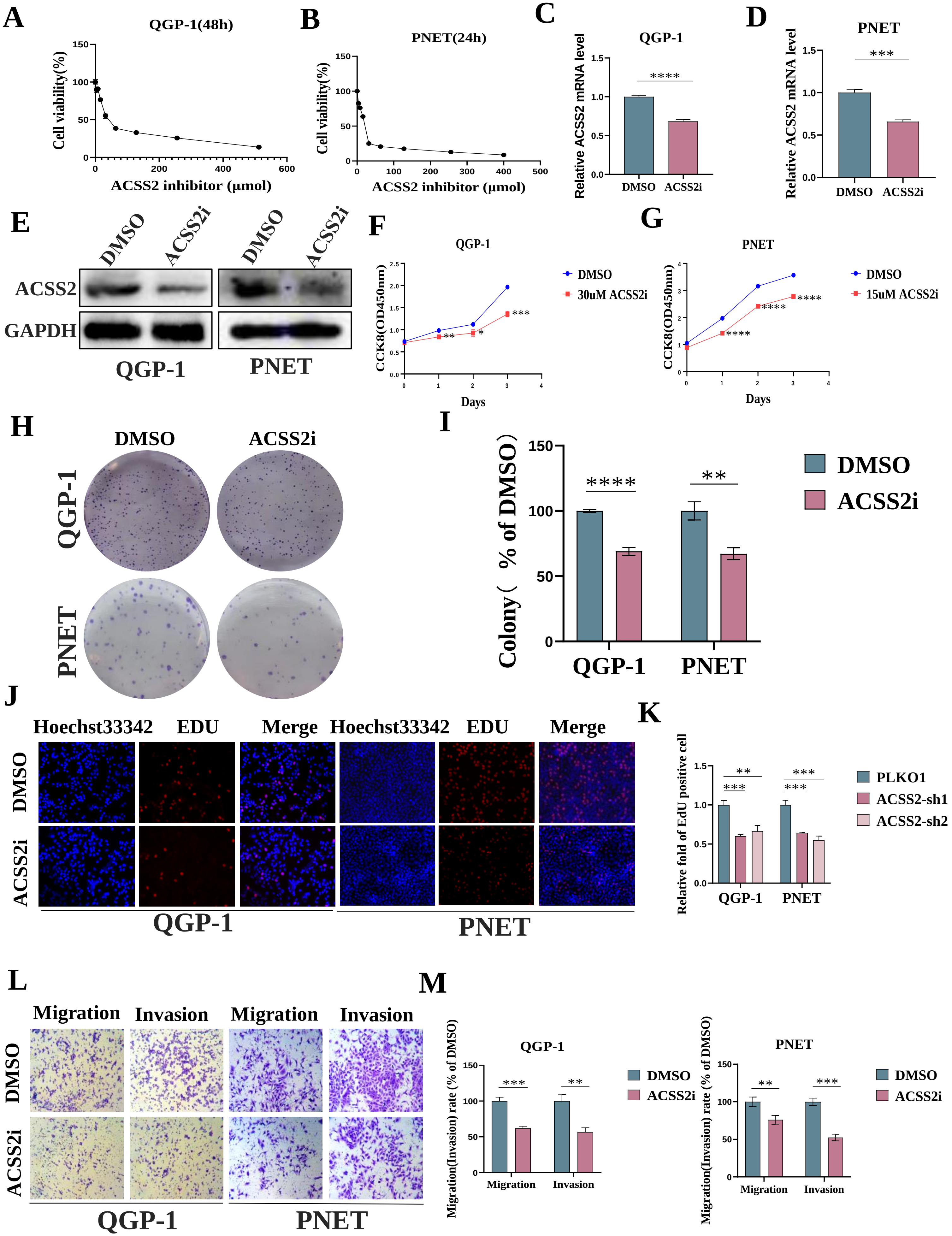

A
QGP-1(48h)
Cell viability(%)
ACSS2 inhibitor (μmol)
B
PNET(24h)
Cell viability(%)
ACSS2 inhibitor (μmol)
C
QGP-1
Relative ACSS2 mRNA level
****
DMSO
ACSS2i
D
PNET
Relative ACSS2 mRNA level
***
DMSO
ACSS2i
E
DMSO
ACSS2i
DMSO
ACSS2i
ACSS2
GAPDH
QGP-1
PNET
F
QGP-1
CCK8(OD450nm)
Days
DMSO
30uM ACSS2i
G
PNET
CCK8(OD450nm)
Days
DMSO
15uM ACSS2i
H
DMSO
ACSS2i
QGP-1
PNET
I
Colony（ % of DMSO）
QGP-1
PNET
DMSO
ACSS2i
J
Hoechst33342
EDU
Merge
Hoechst33342
EDU
Merge
DMSO
ACSS2i
QGP-1
PNET
K
Relative fold of EdU positive cell
QGP-1
PNET
PLKO1
ACSS2-sh1
ACSS2-sh2
L
Migration
Invasion
Migration
Invasion
DMSO
ACSS2i
QGP-1
PNET
M
QGP-1
Migration(Invasion) rate (% of DMSO)
Migration
Invasion
DMSO
ACSS2i
PNET
Migration(Invasion) rate (% of DMSO)
Migration
Invasion
DMSO
ACSS2i

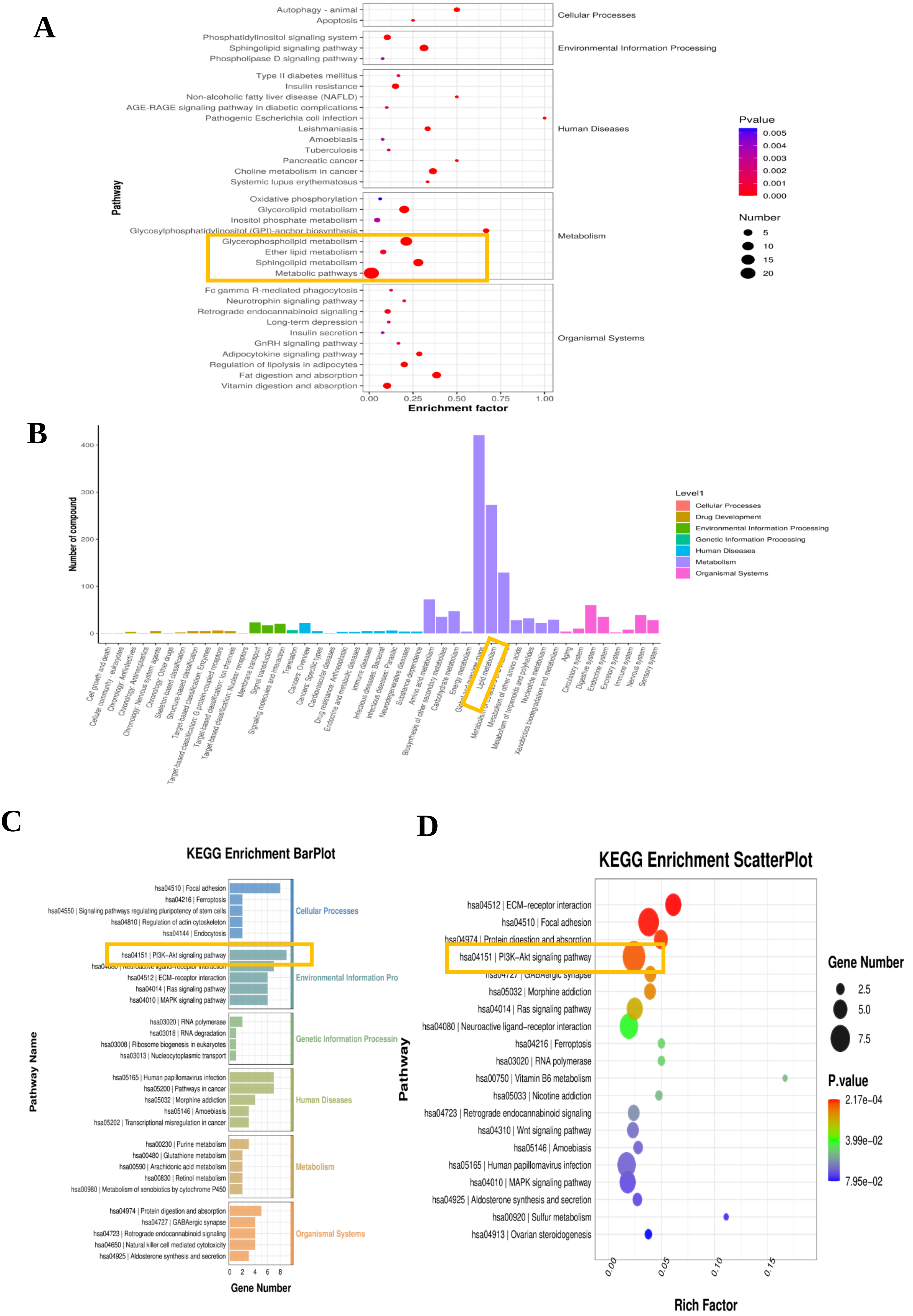
A
Autophagy - animal
Apoptosis
Cellular Processes
Phosphatidylinositol signaling system
Sphingolipid signaling pathway
Phospholipase D signaling pathway
Environmental Information Processing
Type II diabetes mellitus
Insulin resistance
Non-alcoholic fatty liver disease (NAFLD)
AGE-RAGE signaling pathway in diabetic complications
Pathogenic Escherichia coli infection
Leishmaniasis
Amoebiasis
Tuberculosis
Pancreatic cancer
Choline metabolism in cancer
Systemic lupus erythematosus
Human Diseases
Oxidative phosphorylation
Glycerolipid metabolism
Inositol phosphate metabolism
Glycosylphosphatidylinositol (GPI)-anchor biosynthesis
Glycerophospholipid metabolism
Ether lipid metabolism
Sphingolipid metabolism
Metabolic pathways
Metabolism
Fc gamma R-mediated phagocytosis
Neurotrophin signaling pathway
Retrograde endocannabinoid signaling
Long-term depression
Insulin secretion
GnRH signaling pathway
Adipocytokine signaling pathway
Regulation of lipolysis in adipocytes
Fat digestion and absorption
Vitamin digestion and absorption
Organismal Systems
Pathway
0.00
0.25
0.50
0.75
1.00
Enrichment factor
Pvalue
0.005
0.004
0.003
0.002
0.001
0.000
Number
5
10
15
20
B
Number of compound
0
100
200
300
400
Level1
Cellular Processes
Drug Development
Environmental Information Processing
Genetic Information Processing
Human Diseases
Metabolism
Organismal Systems
Cell growth and death
Cellular community - eukaryotes
Chronology: Antiinfectives
Chronology: Antineoplastics
Chronology: Nervous system agents
Chronology: Other drugs
Skeleton-based classification
Structure-based classification
Target-based classification: Enzymes
Target-based classification: G protein-coupled receptors
Target-based classification: Ion channels
Target-based classification: Nuclear receptors
Membrane transport
Signal transduction
Signaling molecules and interaction
Translation
Cancers: Overview
Cancers: Specific types
Cardiovascular diseases
Drug resistance: Antineoplastic
Endocrine and metabolic diseases
Immune diseases
Infectious diseases: Bacterial
Infectious diseases: Parasitic
Neurodegenerative diseases
Substance dependence
Amino acid metabolism
Biosynthesis of other secondary metabolites
Carbohydrate metabolism
Energy metabolism
Lipid metabolism
Metabolism of other amino acids
Metabolism of terpenoids and polyketides
Nucleotide metabolism
Xenobiotics biodegradation and metabolism
Aging
Circulatory system
Digestive system
Endocrine system
Excretory system
Immune system
Nervous system
Sensory system
C
KEGG Enrichment BarPlot
hsa04510 | Focal adhesion
hsa04216 | Ferroptosis
hsa04550 | Signaling pathways regulating pluripotency of stem cells
hsa04810 | Regulation of actin cytoskeleton
hsa04144 | Endocytosis
Cellular Processes
hsa04151 | PI3K–Akt signaling pathway
hsa04512 | ECM–receptor interaction
hsa04014 | Ras signaling pathway
hsa04010 | MAPK signaling pathway
Environmental Information Pro
hsa03020 | RNA polymerase
hsa03018 | RNA degradation
hsa03008 | Ribosome biogenesis in eukaryotes
hsa03013 | Nucleocytoplasmic transport
Genetic Information Processin
hsa05165 | Human papillomavirus infection
hsa05200 | Pathways in cancer
hsa05032 | Morphine addiction
hsa05146 | Amoebiasis
hsa05202 | Transcriptional misregulation in cancer
Human Diseases
hsa00230 | Purine metabolism
hsa00480 | Glutathione metabolism
hsa00590 | Arachidonic acid metabolism
hsa00830 | Retinol metabolism
hsa00980 | Metabolism of xenobiotics by cytochrome P450
Metabolism
hsa04974 | Protein digestion and absorption
hsa04727 | GABAergic synapse
hsa04723 | Retrograde endocannabinoid signaling
hsa04650 | Natural killer cell mediated cytotoxicity
hsa04925 | Aldosterone synthesis and secretion
Organismal Systems
Pathway Name
0
2
4
6
8
Gene Number
D
KEGG Enrichment ScatterPlot
hsa04512 | ECM–receptor interaction
hsa04510 | Focal adhesion
hsa04974 | Protein digestion and absorption
hsa04151 | PI3K–Akt signaling pathway
hsa05032 | Morphine addiction
hsa04014 | Ras signaling pathway
hsa04080 | Neuroactive ligand–receptor interaction
hsa04216 | Ferroptosis
hsa03020 | RNA polymerase
hsa00750 | Vitamin B6 metabolism
hsa05033 | Nicotine addiction
hsa04723 | Retrograde endocannabinoid signaling
hsa04310 | Wnt signaling pathway
hsa05146 | Amoebiasis
hsa05165 | Human papillomavirus infection
hsa04010 | MAPK signaling pathway
hsa04925 | Aldosterone synthesis and secretion
hsa00920 | Sulfur metabolism
hsa04913 | Ovarian steroidogenesis
Pathway
0.00
0.05
0.10
0.15
Rich Factor
Gene Number
2.5
5.0
7.5
P.value
2.17e−04
3.99e−02
7.95e−02
