# Supplementary material for: Hypoxia upregulating ACSS2 enhances lipid metabolism reprogramming through HMGCS1 mediated PI3K/AKT/mTOR pathway to promote the progression of pancreatic neuroendocrine neoplasms
Source: J Transl Med. 2024 Jan 23;22:93. doi: 10.1186/s12967-024-04870-z (PMC10804556; doi:10.1186/s12967-024-04870-z)
Supplement: Supplementary file 2 — Additional file 2: Table S1. Primers and shRNA used in the study. Table S2. Antibodies used in the study. [file 12967_2024_4870_MOESM2_ESM.docx]

**Table S1.Primers and shRNA used in the study.**

| Gene | Sequences |
| --- | --- |
| ACSS2 | Forward 5'-AAAGGAGCAACTACCAACATCTG-3' |
|  | Reverse 5'-GCTGAACTGACACACTTGGAC-3' |
| HIF-1a | Forward 5'-GCAAGCCCTGAAAGCG-3' |
|  | Reverse 5'-GGCTGTCCGACTTTGA-3' |
| β-Actin | Forward 5'-CATGTACGTTGCTATCCAGGC-3' |
|  | Reverse 5'-CTCCTTAATGTCACGCACGAT-3' |
| HMGCS1 | Forward 5'-AAGTCACACAAGATGCTACACCG-3' |
|  | Reverse 5'-TCAGCGAAGACATCTGGTGCCA-3' |
| ACSS2-sh1 | 5'-AAGGAGCAACTACCAACATCT-3' |
| ACSS2-sh2 | 5'-GTGGAGCATTGTGGACAAATA-3' |
| HIF-1a-sh1 | 5'-CCAGTTATGATTGTGAAGTTA-3' |
| HIF-1a-sh2 | 5'- GTGATGAAAGAATTACCGAAT-3' |

**Table S2. Antibodies used in the study.**

| Antibody | Catalog# | Working concentration | Manufacturer |
| --- | --- | --- | --- |
| ACSS2 | ab133664 | 1:1000 | Abcam |
| HIF-1a | 3434S | 1:1000 | CST |
| β-Actin | 81115-1-RR | 1:5000 | Proteintech |
| GAPDH | 10494-1-AP | 1:5000 | Proteintech |
| HMGCS1 | 17643-1-AP | 1:1000 | Proteintech |
| PI3K | 20584-1-AP | 1:1000 | Proteintech |
| pAKT | 4060S | 1:1000 | CST |
| AKT | 9272S | 1:1000 | CST |
| pmTOR | ab109268 | 1:1000 | Abcam |
| mTOR | ab134903 | 1:1000 | Abcam |
